# Mini review: Ethnopharmacology and phytochemistry of the tropical American family Marcgraviaceae

**DOI:** 10.3389/fphar.2025.1622814

**Published:** 2025-07-21

**Authors:** Ana Francis Carballo-Arce, Luis Roberto Villegas-Peñaranda, Raúl Esteban Garro-Álvarez, Yohana Alfaro-Ureña, Manuel Sandoval-Barrantes, Martha Mullally, John Thor Arnason

**Affiliations:** ^1^ Biorefinery Research and Innovation Center, CIIBio. Universidad Nacional, Heredia, Costa Rica; ^2^ Department of Biology, Carleton University, Ottawa, Canada; ^3^ Department of Biology, University of Ottawa, Ottawa, Canada

**Keywords:** Marcgraviaceae, anxiolytics, antiviral, antileischmanial, biofilm inhibitors, triterpenes, napthaquinones

## Abstract

The Marcgraviaceae is a neotropical family of lianas and shrubs that has received limited investigation for its medicinal properties. Characterized by prominent, nectar-rich terminal inflorescences, the family comprises 7 genera and 136 species. Traditional uses among Indigenous communities in the Americas include treatments for anxiety, sleep disorders, mental health conditions, and various dermatological ailments. Pharmacological and phytochemical studies have confirmed that extracts from the genus *Marcgravia*, traditionally used for dermatological conditions, inhibit bacterial quorum sensing, with active principles identified as naphthoquinones. *Schwartzia brasilensis* (syn. *Norantea brasilensis*) has demonstrated antiviral activity against Dengue virus, *in vivo* antimalarial efficacy, anti-inflammatory properties, and DNA-protective effects, but active principles remain to be accurately determined. *Ruyschia phylladenia*, containing triterpenes and isofraxidin, has shown promising antileishmanial, antibacterial, and antitumor activities. Pharmacological research on *Souroubea* spp. from Central America has revealed strong anxiolytic properties in animal models, with active compounds identified as the triterpenes betulinic acid, α-amyrin, and β-amyrin. Following toxicity and efficacy trials, *Souroubea sympetala* leaf extracts have been developed into a practical veterinary formulation for the management of noise aversion in dogs. Given the extensive diversity and wide distribution of this tropical American family, Marcgraviaceae offers considerable untapped potential for the discovery of new medicinal properties and phytochemicals.

## 1 Introduction

Marcgraviaceae is a tropical plant family comprising 7 accepted genera and 136 species (POWO, 2025) of lianas, woody epiphytes, and shrubs with recent species discovery and taxonomic study (Dressler, 2000; Giraldo-Canas, 2001; Giraldo-Cañas, 2018; 2023; Giraldo-Cañas et al., 2024). Despite their striking flowers and ecological importance, their phytochemical and pharmacological properties have remained largely unexplored until recent decades. Ethnobotanical evidence indicates that some species are traditionally used for medicinal purposes, a notion supported by phytochemical studies identifying bioactive compounds.

**FIGURE 1:**
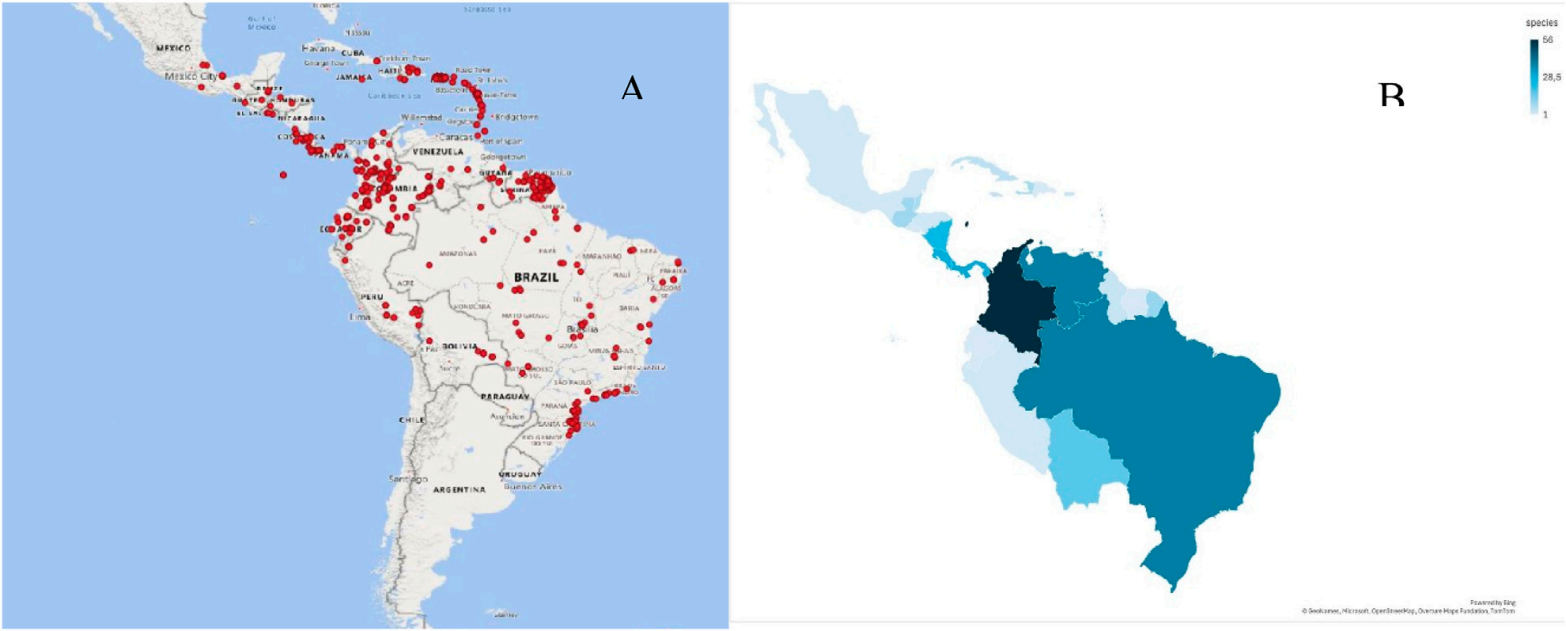
**(A)** Human sightings report of Marcgraviaceae species in Neotropics. Data Source: Global Biodiversity Information Facility, GBIF (Global Biodiversity Information Facility, n.d.). **(B)** Neotropical Graphical representation of Marcgraviaceae species according to Giraldo-Cañas (2018).

The family is distributed (Figure 1) from Mexico to Brazil and across the Caribbean, predominantly in rainforest habitats up to 1,500 m in elevation and with greatest frequency in Northwest regions of South America (Dressler, 2004; Giraldo-Cañas, 2018). Its members display racemose or umbellate inflorescences with nectar-producing bracts that attract diverse pollinators, including insects, bats, and occasionally opossums. Many species exhibit vivid red or orange flowers and have ornamental potential. Morphologically, their coriaceous, alternately arranged leaves feature inconspicuous secondary venation and prominent glands; young leaves envelop the apical meristem. The seven accepted genera (number of species) are: *Marcgravia* (62), *Marcgraviastrum* (15), *Norantea* (1), *Souroubea* (20), *Schwartzia* (20) *Sarcopera* (8), and *Ruyschia* (10) (POWO, 2025).

In the current review, we searched the genera of Marcgraviaceae on PubMed, Google Scholar, Scopus and Web of Science to identify peer-reviewed papers published between 1960 and 15 April 2025, that reported botanical information, ethnobotany, phytochemistry and pharmacological activity of Marcgraviaceae. The set of keywords used for the search in the platforms and engines were: Marcgraviaceae, each of the eight genera names, a combination of pharmacology, ethnobotany and phytochemistry. We also assessed some reports in books and dissertations that were available at the University of Ottawa library and online.

## 2 Ethnobotany

Schultes and Raffauf (1990) reported the use of several species of Marcgraviaceae in “the Healing Forest”, which is a compendium of 1,500 historical ethnobotanical records of medicinal and toxic plants for Northwest Amazonia. A warm tea from the leaves and flowers of *Marcgraviastrum elegans* was used by the Kubeo Indigenous people of Colombia to aid elderly individuals with sleep. Flowers of *Norantea guianensis* were crushed and applied to infected skin by Kuripak, Karapana and Taiwano communities along the Rio Apaporis in Brazil. A leaf tea from *Souroubea crassipetala* was used to induce sleep and to treat mouth sores. The Witoto near Leticia prepared a pomade from animal fat and leaves of *Souroubea pachyphylla* as a conjunctivitis treatment, In the Rio Vaupes, *Souroubea guianensis* leaves were boiled by the Kubuyari to produce a drink that calmed nervous elderly patients. The Taiwanos also used leaves of *S. guianensis* for treating facial skin conditions and as a decoction for managing the culture-bound syndrome known as “*susto*”. Traditionally, healers describe *susto* as resulting from a sudden fright that causes the loss of the “soul” or essence (Mullally et al., 2008). Reported symptoms include unease, worry, social withdrawal, sadness, weight loss, and other indicators consistent with folk descriptions of anxiety (Bourbonnais-Spear et al., 2007; Puniani et al., 2014). *Susto* has been linked by the American Psychiatric Association and the World Health Organization (1992) to anxiety and related mental health disorders.

In Central America, ethnobotanical surveys with Q’eqchi’ Maya healers in Belize revealed the use of a decoction of the leaves of *Souroubea sympetala* as treatment for effects of reputed witchcraft, which leads to anxiety symptoms (Puniani et al., 2014). Balick and Arvigo (2015) also reported the use of leaves this species by Q’eqchi’ for treatment of “baysore” (Cutaneous Leischmaniasis).

In eastern South America, other authors (Sousa do Nascimento Monteiro et al., 2024) reported the use of *N. guianensis* for treatment of headaches, intestinal disorders, insect bites, syphilis, nausea, ulcerative wounds, and fever. Dressler (2004) reported that other genera of Marcgraviaceae, including *Marcgravia*, *Norantea*, *Sarcopera*, and *Souroubea*, were traditionally used among various South American ethnic groups for managing headaches, toothaches, insect bites, diarrhea, and syphilitic wounds.

Little information is available regarding the traditional use of *Marcgraviastrum* and *Sarcopera* and none for *Ruyschia*. The only food use is an ethnobotanical inventory of lianas of Amazonian Ecuador listed *Margravia* sp. as food (Paz et al., 1995). In addition, we found no reports for the Caribbean in the TRAMIL database (TRAMIL, 2025) or in the ethnobotany of the Shuar of Ecuador (Bennett et al., 2002).

## 3 Phytochemistry

### 3.1 Phenolic compounds and coumarins

**FIGURE 2:**
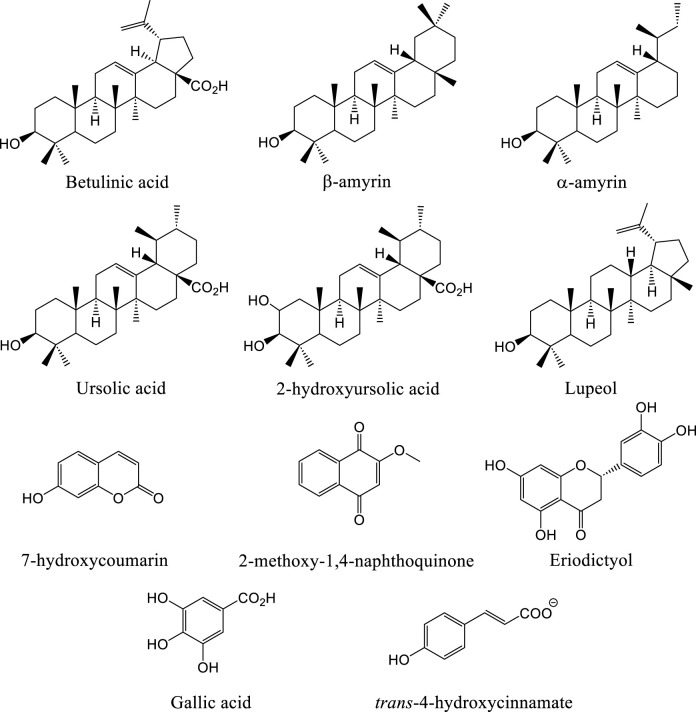
Chemical compounds identified in Marcgraviaceae family.

Two broad reviews of the phytochemistry of the family (Hegnauer, 1996; Rocha, 2002) are part of chemosystematic studies of the order Rutales, and reported flavonols, sterols, triterpenes and naphthoquinones as key compounds in the family. Several flavonol glycosides were reported from the flowers of *N. guianensis*, including myricetin-3-galactoside, myricetin-3-arabinoside, myricetin-3-rhamnoside, quercetin-3-galactoside, quercetin-3-arabinoside, quercetin-3-rhamnoside, and kaempferol-3-galactoside (Saleh and Tower, 1974). Subsequent studies confirmed that *N. guianensis* leaves are rich in phenolic derivatives such as gallic acid, rutin, and quinones, which contribute to allelopathic effects on lettuce and tomato seedlings (Morbeck de Oliveira et al., 2022) (Figure 2). Additionally, coumarins have been identified within the family. For instance, isofraxidin, isolated from the bark of *Ruyschia phylladenia*, exhibited significant antileishmanial activity against *Leishmania amazonensis* (Steinberg et al., 2016). Partial phytochemical analysis of *Souroubea gilgii* leaves revealed the presence of taraxenyl trans-4-hydroxycinnamate and two methylated derivatives of naringenin, as well as a partially methylated flavanone, eriodictyol (Puniani et al., 2014) (Figure 2).

### 3.2 Triterpenes

Further research identified triterpenes as the key compounds responsible for the anxiolytic activity in Marcgraviaceae, highlighting them as the family’s most prominent secondary metabolites (Puniani et al., 2014) (Figure 2). Pentacyclic triterpenoids, particularly betulinic acid (BA), ursolic acid (UA), α-amyrin, and β-amyrin occur in *S. sympetala*, where these compounds show organ-specific distribution. BA and UA are predominantly found in the bark, wood, and flowers, while α- and β-amyrins are concentrated in the leaves (Mullally et al., 2008; 2011; Puniani et al., 2014; Liu et al., 2017; 2019). Similarly, the bark of *R. phylladenia* has yielded lupeol and betulinic acid, both demonstrating cytotoxic activity against human tumor cell lines (Steinberg et al., 2016).

### 3.3 Polyketides

Bioassay-guided fractionation of the ethanolic leaf extract of *Marcgravia nervosa* led to the identification of 2-methoxy-1,4-naphthoquinone (MeONQ), as the compound responsible for biofilm inhibition (Figure 2). This finding represents the first report of a polyketide-type compound within the Marcgraviaceae family (Carballo-Arce et al., 2015). MeONQ has shown anticancer activity related to cytotoxicity and cell necrosis in previous reports (Wang and Lin, 2012).

## 4 Pharmacology

Emerging antimicrobial strategies target bacterial communication through quorum sensing (QS) to curb biofilm formation and the expression of virulence factors (Velasco-Bucheli et al., 2020). Building on this concept, extracts from leaves of 12 species of Marcgraviaceae were screened for their ability to inhibit bacterial quorum sensing (QS) in *Chromobacterium violaceum*, to prevent biofilm formation by *Pseudomonas aeruginosa* PA14, and to inhibit fungal growth in both haploid and diploid accessions of *Saccharomyces cerevisiae* (Carballo-Arce et al., 2015). Ethanolic leaf extracts of three *Marcgravia* spp. (*M*. *nervosa*., *Marcgraviastrum polyantha*, *Marcgraviastrum schippii*), showed significant activity in the QS bioassay at 1 mg·disc^−1^. *M*. *polyantha* extracts significantly inhibited biofilm inhibition at 400 μg mL^−1^ and *M*. *nervosa*, inhibited fungal growth at 2 mg·disc^−1^. Species of the genera *Sarcopera*, *Schwartzia*, *Souroubea* and *Marcgraviatrum* showed some inhibitory trends but were not significant. The leaf extract of *M. nervosa* was subjected to bioassay guided isolation and 2-methoxy-1,4-naphthoquinone was identified as the active principle. It showed potent QS inhibition at the tested concentration of 20 mg·disc^−1^. The minimum inhibitory concentration (MIC) for this compound was determined to be 85 and 100 mol·L^−1^ against *S. cerevisiae* BY4741 (haploid) and BY4743 (diploid).

*Norantea guianensis* was recently investigated for embryotoxicity using a zebrafish (*Danio rerio*) model. The leaf extract exhibited an LC_50_ value of 7.16 mg^.^L^−1^ and demonstrated inhibition of acetylcholinesterase activity at a concentration of 80 mg^.^L^−1^ (Sousa do Nascimento Monteiro et al., 2024).

The crude ethanolic leaf extracts from *Schwartzia brasiliensis* (syn *Norontea brazilensis*) exhibited antiviral activity using a cellular assay against Dengue virus in a concentration-dependent manner at 1 and 10 μg mL^−1^. For immunomodulation, the dichloromethane subfraction showed the greatest reduction in inflammatory cytokines TNF-α and IL-6. Although biologically active subfractions were identified, the active principles remain to be isolated and characterized (Fialho et al., 2017).

A thesis study (Mello, 2012) evaluated the antimalarial activity of an alcoholic extract from *in vitro*-grown roots of *Schwartzia brasilensis*. The extract demonstrated a significant reduction in parasitemia at a dose of 50 mg kg^−1^ in a mouse model infected with *Plasmodium berghei*. In a further study (Mello et al., 2015), the genoprotective effects of alcoholic and aqueous extracts from the leaves and stems of *S. brasilensis* were assessed, demonstrating a reduction in DNA strand breaks induced by stannous chloride at a concentration of 250 μg mL^−1^.

Bark extracts of *R. phylladenia* collected from Monte Verde, Costa Rica, demonstrated promising antiparasitic activity against *L. amazonensis* (Monzote et al., 2014). The extracts exhibited an LC_50_ of less than 12.5 μg mL^−1^ against promastigotes and 22 μg mL^−1^ against amastigotes. Additionally, the extracts showed antimicrobial activity against *Bacillus cereus* and *Staphylococcus aureus*, as well as cytotoxic effects on human tumor cell lines. Bioassay-guided fractionation of the crude acetone bark extract led to the isolation and identification of lupeol, betulinic acid, and isofraxidin. Lupeol and betulinic acid exhibited cytotoxicity against MCF-7, MDA-MB-231, and 5,637 human tumor cell lines, while isofraxidin demonstrated specific antileishmanial activity against *L*. *amazonensis* promastigotes (Steinberg et al., 2016).

Initial studies demonstrated that extracts of *S. sympetala* significantly reduced anxiety-related behaviors in the elevated plus maze (EPM) test using Sprague-Dawley rats (Mullally et al., 2011; Puniani et al., 2014). Alcohol or ethyl acetate extracts of leaf increased, in a dose dependent fashion, the time spent in the open arms of the maze or unprotected head dips, both indications of antianxiety activity. Further studies using the conditioned emotional response (CER) paradigm in rats revealed a dose-dependent reduction in fear-related behaviors (Cayer, 2012). Comparative extraction procedures revealed that supercritical CO_2_ extracts of the leaves exhibited the most significant anxiolytic effects, with activity at 25 mg kg^−1^, comparable to diazepam at 10 mg kg^−1^ (Mullally et al., 2011).

Bioassay-guided isolation led to the identification of betulinic acid (BA), a pentacyclic triterpene, as an active principle. BA demonstrated significant anxiolytic effects in the EPM test, increasing time spent in open arms at a dose of 0.5 mg kg^−1^ administered orally. Furthermore, derivatives such as methyl betulinic acid (Methyl-BA) also exhibited anxiolytic activity in similar animal models. Other triterpenes isolated, including α-amyrin, β-amyrin, and ursolic acid, did not significantly enhance open-arm time individually but may contribute synergistically to the overall anxiolytic effect (Liu et al., 2017). The anxiolytic activity of leaf. extracts of *Souroubea* spp. have been further confirmed in additional models using mice and dogs (Puniani et al., 2014; Liu et al., 2017).

Studies of *Souroubea* spp. leaf extracts or pure BA have identified several mechanisms of action of interest. The anxiolytic effect in rodents appears to be similar to benzodiazepines. Evidence for this comes from pretreatment of rodents with GABA_A_-BZD receptor antagonist, flumazenil, which abolishes the anxiolytic activity (time spent in open arms for rodent model) of extracts of *Souroubea* as well as methyl-BA (Mullally et al., 2014) In addition, these agents can displace labelled GABA_A_-BZD receptor ligands from rat brain preparations.

In trout, treatment with BA resulted in a reduction of plasma cortisol levels in stressed fish, but not in unstressed fish (Mullally et al., 2017). In trout head kidney cell preparations, BA lowered cortisol production in a concentration-dependent manner, suggesting that the cortisol-reducing effect is not mediated through GABA_A_-benzodiazepine (GABA_A_-BZD) receptor binding. Similarly, cortisol reduction by BA has been demonstrated in stressed, but not unstressed, rodents.

Murkar et al. (2019) investigated the effects of *S. sympetala* leaf extract, betulinic acid (BA), and betulin (BE) in a rodent model of fear memory consolidation, which is considered a relevant model for studying post-traumatic stress disorder (PTSD) in humans. The *S. sympetala* leaf extract significantly attenuated the reconsolidation of contextual fear at doses of 25 and 75 mg kg^−1^, but not at 8 mg kg^−1^. Moreover, the combination of BA and BE, but not either compound alone, attenuated the reconsolidation of learned fear and exerted an anxiolytic-like effect on fear expression (Murkar et al., 2019).

These findings supported the development of a commercial formulation to reduce noise aversion in dogs (Liu et al., 2017). To lower production costs, a mixed extract from *S. sympetala* leaves and stems and *Platanus americanus* bark (also rich in BA) was combined with beef flavoring and binders into a chewable tablet. The product proved safe in a pilot study and a 28-day feeding trial in beagles at twice the recommended dose, significantly reducing anxiety behaviors during simulated thunderstorms (Masic et al., 2018; 2021). Methods for propagation and plantation of *S sympetala* have been developed (Vargas et al., 2020).

## 5 Discussion

Despite the Marcgraviaceae family having approximately 136 species, less than ten traditionally used species records were readily retrievable from the peer reviewed literature. Furthermore, the Schultes and Raffauf (1990) compendium from the Northwest Amazon provides only five Marcgraviaceae species records despite 1,500 species reports from over 120 individual collectors. The key word retrieval method has its limitations, but consultation of species records in several major ethnobotanies such as TRAMIL (2025) database or Bennett et al. (2002) yielded no further records. Overall, the ethnobotanical record for this tropical family remains limited and it may be considered a low use family. This likely reflects the rarity or ecological inaccessibility of many species. In fact, most of the records for taxonomy and ecology of these species provide a consensus in reporting their limited distribution and infrequent occurrence (Dressler, 2000; Giraldo-Canas, 2001 Laube and Zotz, 2007). These papers also show the specialized habitats preferred by these species where they often grow in the canopy as hemi epiphytes or vines on large trees (Hammel, 2006).

Detailed phytochemical and pharmacological studies are mostly related to ethnobotanical use and also remain limited, underscoring substantial opportunities for novel discoveries and therapeutic applications if other species are studied. The presence of triterpenes, particularly betulinic acid, ursolic acid, and α- and β-amyrins, constitutes one of the most prominent and well-documented phytochemical features within this family (Puniani et al., 2014). BA extensively studied in *S. sympetala*, demonstrated clear anxiolytic effects in animal models, showing mechanisms of action similar to benzodiazepines, notably involving the modulation of GABA_A_-BZD receptor activity (Mullally et al., 2014; Puniani et al., 2014). The practical translation of these findings into veterinary applications, particularly for noise aversion in dogs, illustrates a viable application pathway. However, market success has been impeded by competition from lower-cost products (Liu et al., 2017; Masic et al., 2018; 2021).

In recent decades naphthoquinones have been identified in *Marcgravia* species exhibit promising QS inhibitory and biofilm-disrupting activities (Carballo-Arce et al., 2015). These findings are particularly relevant given the increasing global challenge of antibiotic resistance and biofilm-associated infections. Nonetheless, research remains in preliminary stages, and further *in vivo* toxicology and efficacy validation studies are necessary before any clinical translation can considered. Similarly, the antiviral, anti-inflammatory, antimalarial, and genoprotective properties of *Schwartzia braziliensis* (syn. *Norantea braziliensis*) show the significant breadth of pharmacological activity of the family (Fialho et al., 2017).

*Ruyschia phylladenia* has shown promising antileishmanial, antimicrobial, and cytotoxic activities. The isolation of compounds like lupeol, betulinic acid, and isofraxidin suggest its medicinal potential (Steinberg et al., 2016). Future investigations should focus on detailed mechanism-of-action studies and *in vivo* validation to progress towards potential therapeutic applications.

## 6 Conclusion

Marcgraviaceae represents an underexplored botanical family with significant potential for the development of anxiolytic, antiparasitic, antimicrobial, and immunomodulatory agents based on published research. Traditional medicinal uses among Indigenous communities, particularly for neurological and inflammatory conditions, show consensus and have guided these targeted pharmacological investigations.

Given the low percentage of species studied, and significant risk to their habitats posed by deforestation and climate change in the neotropics, collection and study of the uninvestigated species is a high priority. Future research should prioritize targeted ethnobotanical surveys and collections in areas where species have been collected previously. These efforts will enable the discovery of new bioactive molecules and the preservation of traditional knowledge before it is lost, thereby contributing to both pharmaceutical development and the conservation of cultural and biological biodiversity. Given the success of previous research on a limited number of species, there is great potential in studying this family.

## References

[B41] Balick M. J. Arvigo R. (2015). Messages from the gods: a guide to the useful plants of Belize. Oxford University Press.

[B1] Bennett B. C. Baker M. A. Andrade P. G. (2002). Ethnobotany of the shuar of eastern Ecuador. Adv. Econ. Bot. 14, i-299. 10.70845/2572-3626.1091

[B2] Bourbonnais-Spear N. Awad R. Merali Z. Maquin P. Cal V. Arnason J. T. (2007). Ethnopharmacological investigation of plants used to treat susto, a folk illness. J. Ethnopharmacol. 109, 380–387. 10.1016/j.jep.2006.08.004 17071033

[B3] Carballo-Arce A. F. Ta C. A. K. Rocha M. E. D. N. Liu R. Harmsen I. Mogg C. D. (2015). Antimicrobial activities of marcgraviaceae species and isolation of a naphthoquinone from *Marcgravia nervosa* (Marcgraviaceae). Botany 93 (7), 413–424. 10.1139/cjb-2015-0038

[B4] Cayer C. (2012). *In vivo* behavioral characterization of anxiolytic botanicals. Canada: University of Ottawa.

[B5] Dressler S. (2000). A new species of *Marcgravia* (Marcgraviaceae) from Amazonia with some notes on the Galeatae group including a key. Willdenowia 30, 369–374. 10.3372/wi.30.30214

[B6] Dressler S. (2004). “Marcgraviaceae,” in Flowering plants. Dicotyledons. Editor Kubitzki K. (Springer), 258–265.

[B7] Fialho L. G. Da Silva V. P. Reis S. R. N. I. Azeredo E. L. Kaplan M. A. C. Figueiredo M. R. (2017). Antiviral and immunomodulatory effects of *Norantea brasiliensis* choisy on Dengue Virus-2. Intervirology 59, 217–227. 10.1159/000455855 28329744

[B8] Giraldo-Canas D. (2001). Dos nuevas especies de *Schwartzia* (Marcgraviaceae) de Colombia. Rev. Acad. Colomb. Cienc. XXV, 477–482. 10.18257/raccefyn.25(97).2001.2779

[B9] Giraldo-Cañas D. (2018). Circunscripción morfológica, diversidad, patrones de distribución y catálogo de la familia neotropical Marcgraviaceae (Ericales). Biota Colomb. 19, 49–69. 10.21068/c2018.v19n01a04

[B10] Giraldo-Cañas D. (2023). Una nueva especie de *Schwartzia* (Marcgraviaceae, Ericales) de Perú. Cinchonia 18, 184–194.

[B11] Giraldo-Cañas D. Trujillo-Trujillo E. Parra-O C. (2024). A new species of *Souroubea* (Marcgraviaceae, Ericales) from Colombia. Rev. Acad. Colomb. Cienc. Exactas Fis. Nat. 48, 298–306. 10.18257/raccefyn.2243

[B12] Hammel B. E. (2006). Three new species of Marcgraviaceae from Costa Rica, with references to related species and notes on the generic placement of *Schwartzia jimenezii* . Lankesteriana 6, 73–81. 10.15517/lank.v6i2.19711

[B13] Hegnauer R. (1996). Chemotaxonomie der Pflanzen. Chemotaxon. Pflanz. 10.1007/978-3-0348-9175-2

[B14] Laube S. Zotz G. (2007). A metapopulation approach to the analysis of long‐term changes in the epiphyte vegetation on the host tree *Annona glabra* . J. Veg. Sci. 18 (5), 613–624. 10.1111/j.1654-1103.2007.tb02575.x

[B15] Liu R. Ahmed F. Cayer C. Mullally M. Carballo A. F. Rojas M. O. (2017). New botanical anxiolytics for use in companion animals and humans. AAPS J. 19, 1626–1631. 10.1208/s12248-017-0144-y 28895076

[B16] Liu R. Carballo-Arce A.-F. Singh R. Saleem A. Rocha M. Mullally M. (2019). A selective ion HPLC-APCI-MS method for the quantification of pentacyclic triterpenes in an anxiolytic botanical dietary supplement for the animal health market. Nat. Prod. Commun. 14, 11–14. 10.1177/1934578x1901400104

[B17] Masic A. Landsberg G. Milgram B. Merali Z. Durst T. Vindas P. S. (2021). Efficacy of *Souroubea-Platanus* dietary supplement containing triterpenes in beagle dogs using a thunderstorm noise-induced model of fear and anxiety. Molecules 26, 2049. 10.3390/molecules26072049 33916654 PMC8038379

[B18] Masic A. Liu R. Simkus K. Wilson J. Baker J. Sanchez P. (2018). Safety evaluation of a new anxiolytic product containing botanicals *Souroubea* spp. and *Platanus* spp. in dogs. Can. J. Veterinary Res. 82 (PMC5764046), 3–11. PMC5764046 29382964

[B19] Mello G. da S. (2012). Avaliação do potencial antimalárico de Norantea brasiliensis Choisy (Marcgraviaceae) cultivada *in vitro* e *in vivo* . Available online at: http://www.bdtd.uerj.br/handle/1/7929.

[B20] Mello G. S. De Mattos J. C. P. Amaral A. C. F. Amorim L. M. F. Caldeira-de-Araujo A. Albarello N. (2015). Assessment of the genotoxic and antigenotoxic potential of crude extracts and fractions of *Schwartzia brasiliensis* (Choisy) Bedell ex Giraldo-Caas. J. Med. Plants Res. 9, 223–230. 10.5897/JMPR2014.5715

[B21] Monzote L. Piñón A. Setzer W. (2014). Antileishmanial potential of tropical rainforest plant extracts. Medicines 1, 32–55. 10.3390/medicines1010032 28933376 PMC5532977

[B22] Morbeck de Oliveira A. K. Matias R. Lacerda Pereira K. C. Rizzi E. S. (2022). Phytotoxic substances with allelopathic activity of *Norantea guianensis* on cultivated species and weeds. Concilium 22, 107–121. 10.53660/clm-398-512

[B23] Mullally M. Cayer C. Kramp K. Rojas M. O. Vindas P. S. Garcia M. (2014). Souroubea sympetala (Marcgraviaceae): a medicinal plant that exerts anxiolysis through interaction with the GABA_A_ benzodiazepine receptor. Can. J. Physiol. Pharmacol. 92, 758–764. 10.1139/cjpp-2014-0213 25140794

[B24] Mullally M. Kramp K. Cayer C. Saleem A. Ahmed F. McRae C. (2011). Anxiolytic activity of a supercritical carbon dioxide extract of *Souroubea sympetala* (Marcgraviaceae). Phytotherapy Res. 25, 264–270. 10.1002/ptr.3246 20648677

[B25] Mullally M. Kramp K. Saleem A. Rojas M. O. Vindas P. S. Garcia M. (2008). Characterization and quantification of triterpenes in the neotropical medicinal plant *Souroubea sympetala* (Marcgraviaceae) by HPLC-APCI-MS. Nat. Product. Commun. 3 (11), 1934578X0800301118. 10.1177/1934578x0800301118

[B26] Mullally M. Mimeault C. Otárola Rojas M. Sanchez Vindas P. Garcia M. Poveda Alvarez L. (2017). A botanical extract of *Souroubea sympetala* and its active principle, betulinic acid, attenuate the cortisol response to a stressor in rainbow trout, Oncorhynchus mykiss. Aquaculture 468, 26–31. 10.1016/j.aquaculture.2016.09.040

[B27] Murkar A. Cayer C. James J. Durst T. Arnason J. T. Sanchez-Vindas P. E. (2019). Extract and active principal of the neotropical vine *Souroubea sympetala* Gilg. block fear memory reconsolidation. Front. Pharmacol. 10, 1496. 10.3389/fphar.2019.01496 31956309 PMC6951415

[B28] Paz Y. M. C. G. Balslev H. Valencia R. (1995). Useful lianas of the Siona-Secoya Indians from Amazonian Ecuador. Econ. Bot. 49, 269–275. 10.1007/BF02862346

[B29] POWO (2025). “Plants of the world online,” in Facilitated by the royal botanic gardens (Kew). Available online at: https://powo.science.kew.org/cite-us (Accessed June 24, 2025).

[B30] Puniani E. Cayer C. Kent P. Mullally M. Sánchez-Vindas P. Poveda Álvarez L. (2014). Ethnopharmacology of *Souroubea sympetala* and *Souroubea gilgii* (Marcgraviaceae) and identification of betulinic acid as an anxiolytic principle. Phytochemistry 113, 73–78. 10.1016/j.phytochem.2014.02.017 24641939

[B31] Rocha M. E. N. (2002). Potencialidades biodinâmicas de Norantea brasiliensis Choisy (Marcgraviaceae). Rio de Janeiro: Doctoral dissertation, Tese Mestrado, Instituto Oswaldo Cruz.

[B32] Saleh N. A. M. Tower G. H. N. (1974). Flavonol glycosides of *Norantea guianensis* flowers. Phytochemistry 13 (9), 2012. 10.1016/0031-9422(74)85149-6

[B33] Schultes R. E. Raffauf R. F. (1990). The healing forest: medicinal and toxic plants of the Northwest Amazonia, 484. 10.1055/s-2006-960193 17226198

[B34] Sousa do Nascimento Monteiro L. Matias R. Fernandes C. E. Otsubo Jaques J. A. Brito I. L. Morbeck de Oliveira A. K. (2024). Evaluation of chemical constituents in *Norantea guianensis* aubl. Extracts, embryotoxicity, and acetylcholinesterase inhibitory potential in *Danio rerio* . Toxicon 251, 108132. 10.1016/j.toxicon.2024.108132 39433259

[B35] Steinberg K. M. Shrestha S. Dosoky N. S. Monzote L. Piñón A. Haber W. A. (2016). Cytotoxic and antileishmanial components from the bark extract of *Ruyschia phylladenia* from Monteverde, Costa Rica. Nat. Prod. Commun. 12, 1–2. 10.1177/1934578X1701200101 30549810

[B36] TRAMIL (2025). Available online at: https://tramil.net (Accessed April 15, 2025).

[B37] Vargas A. R. Gomez A. H. Liu R. Rojas M. O. Vindas P. S. Durst T. (2020). *In vitro* culture of the new anxiolytic plant, *Souroubea Sympetal*a. J. Nat. Health Prod. Res. 2, 1–10. Available online at: https://jnhpresearch.com/index.php/jnhpr/article/view/7 (Accessed April 17, 2025).

[B38] Velasco-Bucheli R. Hormigo D. Fernández-Lucas J. Torres-Ayuso P. Alfaro-Ureña Y. Saborido A. I. (2020). Penicillin acylase from *Streptomyces lavendulae* and Aculeacin A acylase from *Actinoplanes utahensis*: two versatile enzymes as useful tools for quorum quenching processes. Catalyst 10, 730. 10.3390/catal10070730

[B39] Wang Y. C. Lin Y. H. (2012). Anti-gastric adenocarcinoma activity of 2-Methoxy-1,4-naphthoquinone, an anti-*Helicobacter pylori* compound from *Impatiens balsamina* L. Fitoterapia 83, 1336–1344. 10.1016/J.FITOTE.2012.04.003 22516543

[B40] World Health Organization (1992). The ICD-10 classification of mental and behavioural disorders: clinical descriptions and diagnostic guidelines, 1. Available online at: https://www.who.int/publications/i/item/9241544228.

